# Efficacy and safety of acupuncture-related therapies in the treatment of allergic rhinitis: a systematic review and meta-analysis

**DOI:** 10.3389/fmed.2026.1835311

**Published:** 2026-05-29

**Authors:** Jun Ma, Meng Peng, Yan-ge Song, Xue-jiao Xu

**Affiliations:** Heilongjiang University of Chinese Medicine, Harbin, Heilongjiang, China

**Keywords:** acupuncture, acupuncture therapy, allergic rhinitis, meta-analysis, systematic review

## Abstract

**Background:**

Acupuncture therapy, as a complementary and alternative approach for allergic rhinitis (AR), has received increasing attention. However, robust conclusions regarding its effectiveness and safety, either as a standalone treatment or in combination with conventional medication, are still lacking. To systematically assess the effectiveness and safety of acupuncture-related therapies in treating AR, a meta-analysis will be performed. This study is designed to directly compare the three primary interventions: acupuncture therapy plus medication, acupuncture therapy alone, and conventional medication therapy, thereby generating evidence-based recommendations for clinical practice.

**Methods:**

We searched relevant reports published in multiple scientific databases and related registration platforms from January 1, 2014 to December 31, 2025, including medical databases such as PubMed, Embase, the Cochrane Library, CNKI, Wanfang, and VIP. The search process was conducted in both Chinese and English. Randomized controlled trials (RCTs) that compared acupuncture therapy with conventional medication, or acupuncture therapy plus conventional medication with conventional medication for patients with AR were included. Two researchers independently extracted data from the included studies using a standardized form and cross-verified their results upon completion to ensure accuracy. For continuous outcomes, effect sizes were calculated as either the standardized mean difference (SMD) or mean difference (MD), both with 95% confidence intervals (CI). For dichotomous outcomes, risk ratios with 95% CI were used. All statistical meta-analyses, subgroup analyses, and assessment of publication bias were performed using RevMan 5.4 software.

**Results:**

This meta-analysis included 38 RCTs involving 3,349 participants. The results showed that, compared with conventional medication alone, both acupuncture therapy alone (SMD = −0.65, 95% CI [−1.17, −0.14]) and acupuncture therapy combined with conventional medication (SMD = −1.18, 95% CI [−1.84, −0.51]) reduced the Total Nasal Symptom Score (TNSS) at the end of treatment. Additionally, improvements were observed in Quality of Life Questionnaire (RQLQ) score (acupuncture therapy: SMD = −1.16, 95% CI [−1.42, −0.90]; combined therapy: SMD = −1.40, 95% CI [−1.95, −0.86]). The reported adverse effects associated with acupuncture therapy were mild and transient. These findings suggest that acupuncture-related therapies demonstrate significant improvements in key outcome measures and may represent an effective and safe approach for treating AR, although the quality of evidence remains low.

**Conclusion:**

Acupuncture-related therapies have shown promising effectiveness in treating AR, effectively alleviating symptoms, improving quality of life, and causing only mild adverse reactions. However, the quality of evidence for certain outcome indicators in the included studies remains low. It is recommended that further validation be conducted through large-scale, multi-center, high-quality RCTs to provide higher-level evidence-based medical support.

**Systematic review registration:**

https://www.crd.york.ac.uk/PROSPERO/view/CRD420251160569, identifier CRD420251160569.

## 1 Introduction

Allergic Rhinitis (AR) is a chronic non-infectious inflammatory disease of the nasal mucosa mediated primarily by Immunoglobulin E (IgE) after exposure to allergens (1). Based on the timing and nature of allergen exposure, AR is clinically classified into two primary forms: Perennial Allergic Rhinitis (PAR) and Seasonal Allergic Rhinitis (SAR). PAR is triggered by year-round indoor allergens such as house dust mites, pet dander, cockroach residues, and mold spores, leading to persistent symptoms throughout the year. In contrast, SAR is typically induced by outdoor pollens from trees, grasses, and weeds, occurring during specific seasons. Its core clinical manifestations include paroxysmal nasal itching, consecutive sneezing, nasal congestion, and watery rhinorrhea, often accompanied by symptoms such as hyposmia, ocular itching, and throat itching (2). The global prevalence of AR remains high, with studies indicating a worldwide prevalence rate of 40% (3) and affecting over 600 million people (4). In recent years, the incidence of AR in China has also risen significantly, with the adult prevalence rate increasing by 6.6% between 2005 and 2011 alone (5), particularly in more industrialized urban areas (6). Although not life-threatening, the recurrent and persistent symptoms of AR severely impact patients’ daily work, academic performance, and sleep quality, and can lead to psychological issues such as mental stress, anxiety, and depression, imposing a substantial health and economic burden on both individuals and society (7). More importantly, AR is a high-risk factor for various complications. If not properly controlled, it can lead to asthma, sinusitis, nasal polyps, otitis media, conjunctivitis, and atopic dermatitis, posing serious threats to patient health (8).

Currently, the primary approaches for the management of AR in modern medicine include allergen avoidance, patient education, pharmacotherapy, and allergen-specific immunotherapy (9). First-line pharmacological treatments include intranasal corticosteroids and second-generation antihistamines. While they can rapidly suppress symptoms and demonstrate good short-term safety (10, 11), issues such as short duration of efficacy, tendency for symptom recurrence, and uncertain long-term effectiveness remain. For approximately 30% of patients with moderate-to-severe AR, symptoms remain difficult to control effectively even with optimal Western medical regimens (12). Furthermore, long-term medication use carries limitations like relatively high costs and potential adverse effects (13, 14), while immunotherapy is constrained by its long treatment duration and high expense. Consequently, finding a treatment method with stable efficacy, good safety, and simple application has become a key focus of current research. Against this backdrop, Traditional Chinese Medicine (TCM) therapies, particularly acupuncture, have gained widespread clinical recognition due to their unique advantages of minimal adverse effects, safety, and high efficacy (15). Numerous clinical studies have reported the significant therapeutic effect of acupuncture in treating AR (16–18). Mechanistic research suggests that acupuncture can regulate the imbalanced Th1/Th2 cell levels in AR patients, reduce eosinophil infiltration, and lessen epithelial damage, thereby protecting the nasal mucosa and addressing the root cause of the disease (19). Supported by substantial evidence, the clinical practice guideline for AR published by the American Academy of Otolaryngology–Head and Neck Surgery has incorporated acupuncture, recommending it for controlling symptoms in patients with perennial AR and improving quality of life, noting that no adverse events related to acupuncture were reported in the guidelines (20).

In summary, acupuncture demonstrates definite efficacy and a favorable safety profile in treating AR, potentially compensating for the lack of specific curative methods and the significant side effects associated with modern medical treatments for this condition (21). However, the current landscape of related clinical research is vast, and their conclusions require synthesis and validation using higher-level evidence. Therefore, this study aims to systematically evaluate the clinical effectiveness and safety of acupuncture-related therapies for AR through Meta-analysis, intending to provide more reliable evidence-based medical support for clinical practice.

## 2 Methods

This study was designed and performed in compliance with the Preferred Reporting Items for Systematic Reviews and Meta-Analyses Protocols (PRISMA-P) statement (22). The trial protocol was prospectively registered in the PROSPERO international prospective register of systematic reviews (Registration ID: CRD420251160569).

### 2.1 Search strategy

To comprehensively identify relevant literature, this study searched the following databases: PubMed, Embase, the Cochrane Library, CNKI, Wanfang, and VIP, with the search period set from January 1, 2014 to December 31, 2025. A systematic search was conducted for RCTs investigating acupuncture-related therapies combined with conventional medication for AR. Search terms included “acupuncture therapy,” “allergic rhinitis,” “randomized controlled trial,” along with relevant synonyms and variants. The search strategy incorporated both subject headings and free-text terms, using the Boolean operators “AND” and “OR.” The detailed search strategy for PubMed is presented in Table 1.

**TABLE 1:** Search strategy used in PubMed for acupuncture-related therapies in allergic rhinitis.

Number	Search terms
#1	[Mesh]: “Rhinitis, Allergic”
#2	[Title/Abstract]: “Allergic Rhinitides” OR “Rhinitides, Allergic” OR “Allergic Rhinitis”
#3	#1 OR #2
#4	[Mesh]: “Acupuncture” OR “Acupuncture Therapy”
#5	[Title/Abstract]: “Pharmacopuncture” OR “Acupuncture Treatment” OR “Acupuncture Treatments” OR “Acupotomies” OR “Acupotomy” OR “Therapy, Pharmacoacupuncture” OR “Pharmacoacupuncture Therapy” OR “Treatment, Pharmacoacupuncture” OR “Pharmacoacupuncture Treatment” OR “Therapy, Acupuncture” OR “Treatment, Acupuncture”
#6	#4 OR #5
#7	[Publication Type]: “randomized controlled trial”
#8	[Title/Abstract]: “randomized” OR “placebo”
#9	#7 OR #8
#10	#3 AND #6 AND #9

### 2.2 Inclusion criteria

Studies were screened for eligibility using the following criteria: (1) Type of studies: RCTs published in Chinese or English. (2) Participants: patients clinically diagnosed with AR based on internationally or nationally recognized criteria (e.g., the Guidelines for the Diagnosis and Treatment of Allergic Rhinitis). No restrictions were placed on gender, age, disease duration, severity, or subtype. (3) Interventions: the experimental group received acupuncture therapy or acupuncture therapy combined with conventional medication (e.g., antihistamines or intranasal corticosteroids). The control group received conventional medication (e.g., oral loratadine or intranasal budesonide). This study primarily evaluates the effectiveness of acupuncture as an add-on or standalone therapy compared with conventional medication in real-world clinical settings, as sham-controlled studies were not included. (4) Outcome measures: studies were required to report at least one of the following primary or secondary outcomes. The details of the outcome measures were adapted from the study by Bao et al. (23). Primary outcome: improvement in nasal symptoms was assessed using the Total Nasal Symptom Score (TNSS), which evaluates nasal congestion, rhinorrhea, sneezing, and nasal itching on a 5-point scale (total range 0–16). Secondary outcomes: ① Total Non-Nasal Symptom Score (TNNSS) evaluated extra-nasal symptoms (postnasal drip, tearing, itching, orofacial pain, headache), each scored as present (1) or absent (0), total range 0–5; ② Quality of life was assessed using the Rhinoconjunctivitis Quality of Life Questionnaire (RQLQ), a 28-item, 7-domain instrument with each item scored 0–6 (total range 0–168); higher scores indicate poorer quality of life; ③ Clinical efficacy rate was defined as the proportion of patients classified as responding to treatment (e.g., “markedly effective” or “effective”), based on the Diagnosis and Treatment Principles and Recommended Protocol for Allergic Rhinitis (2004, Lanzhou) (24), using the improvement rate (IR) of symptom and sign scores: markedly effective (IR ≥ 66%), effective (25% < IR < 66%), and ineffective (IR ≤ 25%); ④ Immunological parameters focused on changes in serum total IgE levels before and after treatment; means, standard deviations, and sample sizes were extracted for meta-analysis; ⑤ Safety data were collected primarily as the total incidence of adverse events (AEs) reported during the treatment period in each group.

### 2.3 Exclusion criteria

The exclusion criteria were: (1) Non-randomized studies, case reports, and case series. (2) Studies for which the full text was unavailable or which contained critical missing data that could not be obtained by contacting the authors. (3) Studies that involved combinations of acupuncture with other therapies (e.g., bloodletting, cupping, or herbal decoctions) to ensure the purity and comparability of the interventions.

### 2.4 Study selection and data extraction

All records retrieved from the databases were managed using EndNote X9, with duplicate entries removed initially. During the literature screening phase, two reviewers (JM and MP) independently evaluated the titles, abstracts, keywords, and full texts of the records according to the predefined inclusion criteria. Data extraction was performed using a pre-designed standardized Excel form by the same two reviewers independently. The extracted information included the first author, year of publication, study design, sample size, participant characteristics, intervention and control measures, outcome indicators, adverse events, as well as main conclusions and details of acupoint selection. Any disagreements arising between the reviewers during screening or data extraction were resolved by a third reviewer, whose decision was final, to ensure the accuracy of the extracted data. The entire process strictly adhered to the PRISMA guidelines and was documented using a flow diagram.

### 2.5 Study quality assessment

The included studies were independently assessed for methodological quality by two reviewers using the Cochrane Collaboration Risk of Bias Tool (25). This evaluation covered six key domains: random sequence generation, allocation concealment, blinding of participants and outcome assessors, incomplete outcome data, selective reporting, and other bias. Each criterion was classified as low risk, high risk, or unclear risk to systematically evaluate potential impacts on study validity. Any disagreements were resolved through discussion with a third reviewer to reach consensus. RevMan 5.4 software was used to generate charts illustrating the risk of bias.

### 2.6 Statistical analysis

This analysis was performed using RevMan 5.4 software. Risk ratios (RR) with 95% confidence intervals (CI) were used as effect measures for dichotomous variables, while mean difference (MD) or standardized mean difference (SMD) with 95% CI were applied for continuous variables. For pooling effect sizes, a fixed-effect model was used when heterogeneity was not significant (*I*^2^ < 50%); otherwise, a random-effects model was applied. This study primarily employed a random-effects model for analysis. Significant heterogeneity was considered when *I*^2^ ≥ 50%, and its sources were explored through sensitivity analysis or subgroup analysis. Publication bias was assessed using funnel plots.

In the presence of significant heterogeneity, sensitivity analysis was undertaken by excluding trials characterized by high risk of bias or marked clinical/methodological heterogeneity. To ensure transparency, we established a priori exclusion criteria and sequentially removed studies meeting any of the following conditions: studies rated as having a “high risk of bias” in any key domain of the Cochrane Risk of Bias assessment tool (e.g., random sequence generation, allocation concealment, or blinding of outcome assessment), or studies whose exclusion reduced the *I*^2^-value by ≥ 10%, thus identifying them as major contributors to statistical heterogeneity. Subsequently, if high heterogeneity persisted for primary outcomes, subgroup analysis was further performed to reduce heterogeneity and enhance the reliability of the results. A two-sided *P* < 0.05 was considered statistically significant for all meta-analyses, and the robustness of the findings was cross-validated by excluding these influential studies.

## 3 Results

### 3.1 Study selection

**FIGURE 1:**
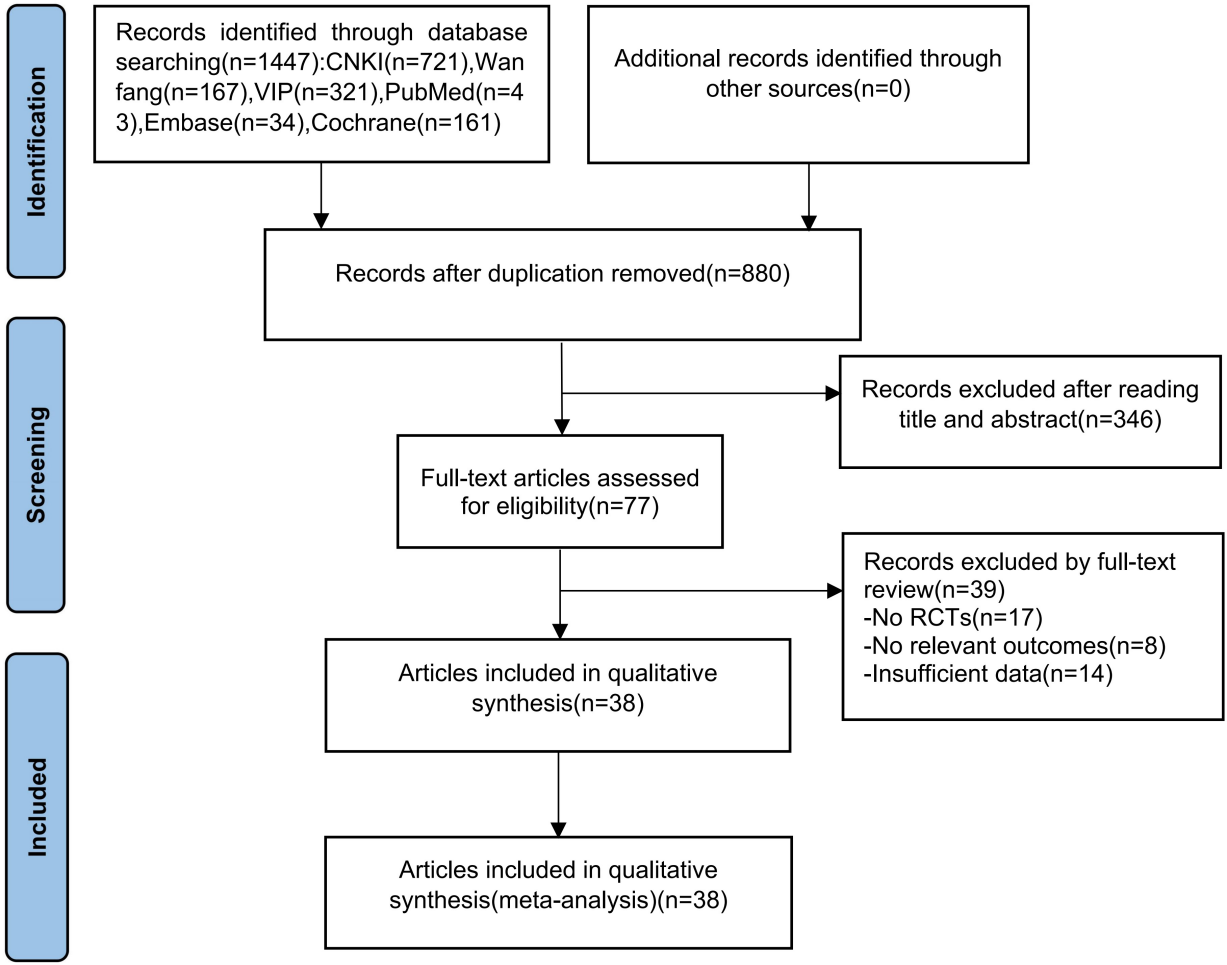
Flow chart of the review process for acupuncture-related therapies in allergic rhinitis.

The literature selection process is summarized in Figure 1. The initial search yielded 1,447 potentially relevant records. After removing 567 duplicates, 880 records underwent title and abstract screening, resulting in 77 articles for full-text assessment. Following a detailed full-text review, 39 articles were excluded for not meeting the eligibility criteria, culminating in 38 RCTs being included in this systematic review.

### 3.2 Characteristics of the included studies

A total of 38 studies (6, 23, 26–61) were included in the final meta-analysis. All of these articles were published between 2014 and 2025, involving 3,349 participants. Participants were systematically allocated to either the experimental group or the control group: the control group received standard Western medical treatment, while the experimental group received acupuncture-related therapies in addition to the control group’s treatment. The sample sizes of the included RCTs ranged from 14 to 154. Group allocation primarily followed a 1:1 ratio, with a nearly balanced male-to-female distribution across studies. Regarding outcome measures, 10 studies (6, 23, 26, 34, 40, 43, 44, 46, 57, 61) reported TNSS; 11 studies (23, 26, 34, 35, 37, 38, 40, 46, 54, 57, 61) documented TNNSS; 17 studies (6, 23, 26–28, 34, 35, 37, 40, 43, 44, 47–49, 54, 58, 61) involved the RQLQ; 36 studies (6, 23, 26–36, 38–60) evaluated clinical efficacy; 17 studies (26–28, 36, 38, 42, 43, 45, 48, 50, 51, 55, 57–61) reported IgE levels; and a total of 9 studies (23, 30, 38, 40, 43, 49, 53, 55, 57) recorded AEs. In terms of acupuncture interventions, a variety of acupoints were selected, primarily including Xinwu point (sphenopalatine ganglion), Yingxiang (LI 20), Yintang (DU 29), Dazhui (DU 14), and Hegu (LI 4). The treatment duration across studies ranged from 2 to 8 weeks, and the frequency of acupuncture interventions ranged from once daily to three times per week, with the most commonly adopted regimens being once every other day or three times per week. Regarding follow-up assessments to evaluate the durability of treatment effects, only 11 of the 38 studies reported follow-up data, with follow-up periods ranging from 4 weeks to 6 months: 3 studies (6, 23, 46) reported a 4-week follow-up, 3 studies (42, 44, 49) reported a 3-month follow-up, and 5 studies (30, 35, 56, 59, 61) reported a 6-month follow-up. Detailed information and findings of all included studies are presented in Table 2.

**TABLE 2:** Characteristics of the included studies on acupuncture-related therapies in allergic rhinitis.

Study	Sample (T/C)	Gender (M:F)	Age (years)	Interventions		Duration of treatment	Outcomes
				T	C		
Bao 2023(23)	40/40	T:19/21 C:22/18	T:36 ± 14 C:35 ± 14	Acupuncture; twice weekly (3–4 days apart)	Budesonide nasal spray	4 weeks	①②③④⑥
Chang 2022 (26)	66/66	T:34/32 C:31/35	T:37.62 ± 6.17 C:36.21 ± 5.44	Acupuncture/umbilical paste+ Loratadine/ Mometasone furoate nasal spray; three times weekly	Loratadine/ Mometasone furoate nasal spray	4 weeks	①②③④⑤
Chen 2015 (6)	34/32	T:17/17 C:14/18	T:44 ± 9 C:40 ± 11	Acupuncture; three times weekly (every other day)	Cetirizine	8 weeks	①③④
Chen 2016 (27)	30/30	T:18/12 C:14/16	T:33 ± 8 C:35 ± 1	Acupuncture+Loratadine/ Mometasone furoate nasal spray; twice weekly	Loratadine/ Mometasone furoate nasal spray	4 weeks	③④⑤
Fang 2018 (28)	25/25	29/21	42	Acupuncture+Loratadine/ Budesonide nasal spray; twice weekly	Loratadine/ Budesonide nasal spray	4 weeks	③④⑤
Feng 2018 (29)	36/35	T:16/20 C:15/20	T:30.3 C:30	Acupuncture; once weekly	Cetirizine	4 weeks	④
Huang 2014 (30)	25/23	T:17/8 C:17/6	T:37.7 ± 7.3 C:36.1 ± 6.1	Acupuncture+Fluticasone propionate nasal spray; every other day	Fluticasone propionate nasal spray	4 weeks	④⑥
Huang 2018 (31)	57/57	T:38/19 C:29/28	T:38.20 ± 6.45 C:37.40 ± 8.12	Acupuncture+Loratadine; once daily	Loratadine	4 weeks	④
Li 2015 (32)	50/50	T:29/21 C:22/28	T:29.45 ± 10.15 C:27.81 ± 11.57	Acupuncture+Loratadine; once daily	Loratadine	4 weeks	④
Li 2016 (33)	25/25	T:13/12 C:15/10	T:32.7 ± 5.1 C:33.2 ± 4.3	Acupuncture+Xiang Ju capsules; once daily	Xiang Ju capsules	3 weeks	④
Li 2018 (34)	45/45	T:25/20 C:27/18	T:35.97 ± 7.47 C:36.09 ± 7.52	Moxibustion+Loratadine/ Budesonide nasal spray; once daily	Loratadine/ Budesonide nasal spray	4 weeks	①②③④
Li 2019 (35)	37/38	T:19/18 C:19/19	T:36.351 ± 2.062 C:38.351 ± 2.307	Acupuncture+Loratadine; once daily	Loratadine	2 weeks	②③④
Li 2021 (36)	28/28	T:14/14 C:13/15	T:41.37 ± 8.69 C:42.81 ± 7.62	Acupuncture+Loratadine; twice weekly	Loratadine	2 weeks	④⑤
Li 2024 (37)	30/30	T:15/15 C:16/14	T:37.87 ± 9.07 C:38.63 ± 9.03	Acupuncture; once daily	Loratadine	2 weeks	②③
Li 2024 (38)	45/45	T:18/27 C:26/19	T:36.6 ± 11.78 C:33.89 ± 10.31	Acupuncture; once daily	Mometasone furoate nasal spray	2 weeks	②④⑤⑥
Liang 2023 (39)	31/31	T:18/13 C:17/14	T:28.12 ± 5.33 C:28.07 ± 5.25	Warm acupuncture+Loratadine; three times weekly (every other day)	Loratadine	8 weeks	④
Liang 2023 (40)	30/30	T:14/16 C:12/18	T:35.3 ± 10.9 C:33.9 ± 11.6	Acupuncture; three times weekly (every other day)	Loratadine	4 weeks	①②③④⑥
Liu 2021 (41)	30/30	T:12/18 C:16/14	T:35.57 ± 8.6 C:33.43 ± 10.3	Acupuncture+Budesonide nasal spray; once daily	Budesonide nasal spray	4 weeks	④
Liu 2021 (42)	30/30	T:16/14 C:17/13	T:36.20 ± 12.11 C:39.23 ± 11.67	Acupuncture; every other day	Loratadine	3 weeks	④⑤
Liu 2022 (43)	40/40	T:25/15 C:21/19	T:32.76 ± 5.73 C:32.15 ± 5.57	Acupuncture+ Mometasone furoate nasal spray; twice weekly	Mometasone furoate nasal spray	4 weeks	①③④⑤⑥
Liu 2023 (44)	32/34	T:15/17 C:17/17	T:34.88 ± 7.29 C:33.32 ± 7.95	Acupuncture+Loratadine/ Budesonide nasal spray; twice weekly (3 days apart)	Loratadine/ Budesonide nasal spray	4 weeks	①③④
Lu 2018 (45)	30/30	/	T:38.65 ± 6.39 C:38.65 ± 6.39	Acupuncture+Ebastine; once daily	Ebastine	4 weeks	④⑤
Luo 2021 (46)	30/30	T:12/18 C:14/16	T:34 ± 8 C:35 ± 7	Acupuncture; three times weekly	Budesonide nasal spray	4 weeks	①②④
Ma 2021 (47)	30/30	T:13/17 C:12/18	T:40.24 ± 8.71 C:40.87 ± 8.46	Warm acupuncture+Loratadine; every other day	Loratadine	4 weeks	③④
Sun 2023 (48)	76/79	T:44/32 C:42/37	T:37.69 ± 6.53 C:39.42 ± 6.68	Warm Acupuncture+Loratadine/ Fluticasone propionate nasal spray; once daily	Loratadine/ Fluticasone propionate nasal spray	2 weeks	③④⑤
Sun 2024 (49)	154/154	T:80/74 C:87/67	T:33.89 ± 4.26 C:34.02 ± 4.34	Warm acupuncture+Fluticasone propionate nasal spray; once daily (5x/week)	Fluticasone propionate nasal spray	4 weeks	③④⑥
Tian 2018 (50)	65/65	T:37/28 C:35/30	T:34.24 ± 1.02 C:33.61 ± 0.94	Acupuncture+Cetirizine; once daily (5x/week)	Cetirizine	4 weeks	④⑤
Wang 2019 (51)	100/100	T:64/36 C:62/38	T:34 ± 9 C:35 ± 9	Warm acupuncture+Loratadine; once daily (5x/week)	Loratadine	2 weeks	④⑤
Wang 2019 (52)	60/60	T:31/29 C:33/27	T:34.9 ± 10.8 C:35.1 ± 10.5	Acupuncture+Fluticasone propionate nasal spray/Azelastine hydrochloride; once daily	Fluticasone propionate nasal spray/Azelastine hydrochloride	4 weeks	④
Wu 2017 (53)	30/30	T: 16/14 C: 17/13	/	Acupuncture+Loratadine/ Budesonide nasal spray; every other day	Loratadine/ Budesonide nasal spray	4 weeks	④⑥
Xia 2022 (54)	32/32	T: 18/14 C: 12/20	T: 39 ± 13 C: 40 ± 12	Acupuncture/ Ginger moxibustion+ Loratadine/ Mometasone furoate nasal spray; three times weekly (every other day)	Loratadine/ Mometasone furoate nasal spray	4 weeks	②③④⑤
Xia 2024 (55)	55/52	T: 23/32 C: 20/32	T:40.29 ± 9.86 C:39.67 ± 10.05	Acupuncture; twice weekly	Fluticasone propionate nasal spray	3 weeks	④⑥
Xu 2015 (56)	55/55	T: 34/21 C: 32/23	T: 36.1 ± 7.4 C: 36.4 ± 7.1	Acupuncture+Tongqiao Biyan particles; once daily	Tongqiao Biyan particles	3 weeks	④
Yang 2019 (57)	42/42	T: 26/16 C: 28/14	T: 41.3 ± 4.9 C: 40.5 ± 7.6	Warm acupuncture+Ebastine/ Budesonide nasal spray; once daily	Ebastine/ Budesonide nasal spray	4 weeks	①②④⑤⑥
Zeng 2020 (58)	26/24	T:11/15 C:10/14	T:41 ± 7 C:40 ± 7	Acupuncture/Acupoint application+Loratadine/ Budesonide nasal spray; once daily	Loratadine/ Budesonide nasal spray	4 weeks	③④⑤
Zhang 2016 (59)	60/60	T: 5/25 C: 38/22	T:36.8 ± 6.1 C:37.3 ± 6.5	Acupuncture+Loratadine; once daily (6x/week)	Loratadine	4 weeks	④⑤
Zhao 2020 (60)	52/51	T: 27/26 C: 24/29	T: 37.31 ± 11.23 C: 38.23 ± 11.19	Acupuncture+Loratadine; once weekly	Loratadine	4 weeks	④⑤
Zheng 2025 (61)	14/14	T: 7/7 C: 6/8	T: 37.85 ± 6.86 C: 37.21 ± 6.53	Acupuncture+ Fluticasone propionate nasal spray; 3x/week (weeks 1–4), 2x/week (weeks 5–6)	Fluticasone propionate nasal spray	6 weeks	①②③⑤

T, Treatment Group; C, Control Group; ①TNSS; ②TNNSS; ③RQLQ; ④Effective rate; ⑤IgE; ⑥AEs.

### 3.3 Risk of bias

**FIGURE 2:**
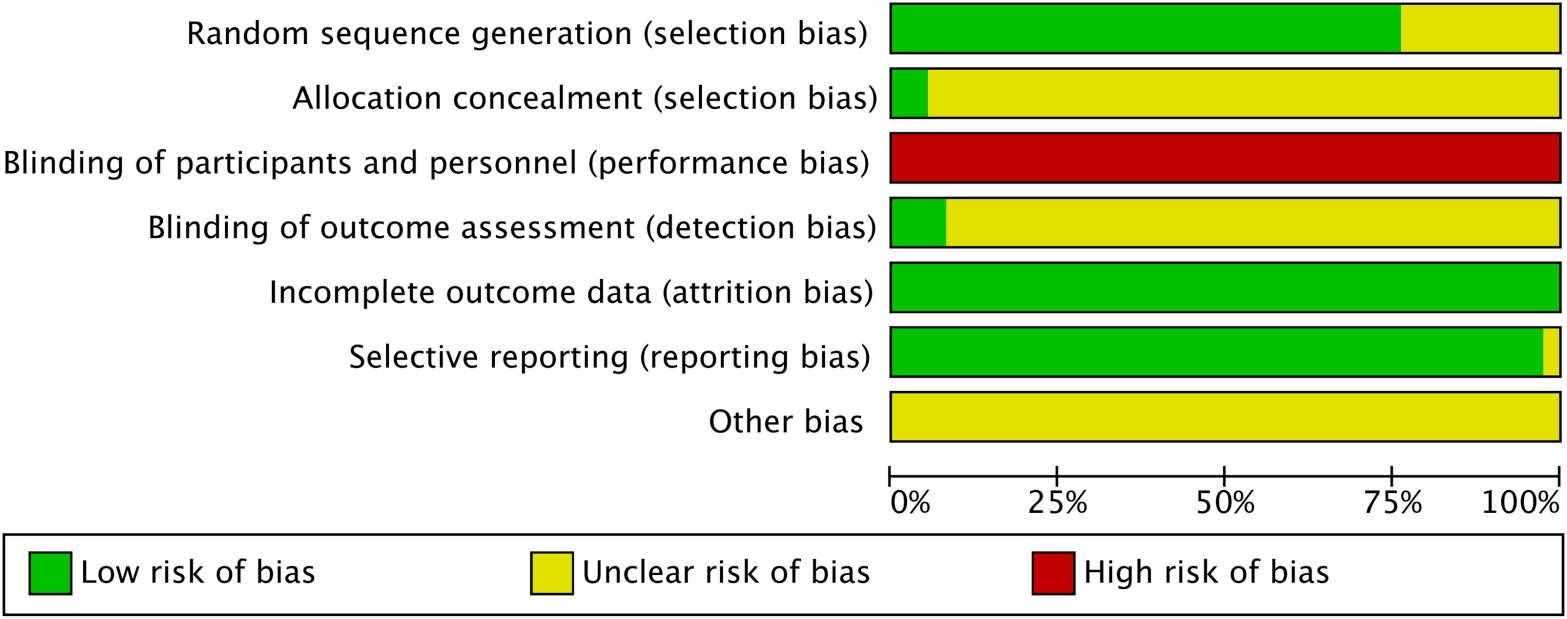
Risk of bias graph for studies on acupuncture-related therapies in allergic rhinitis.

**FIGURE 3:**
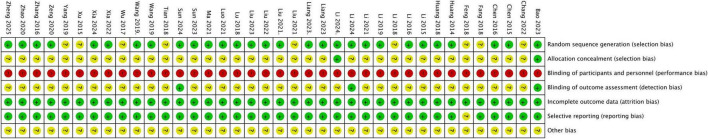
Risk of bias summary for studies on acupuncture-related therapies in allergic rhinitis.

This study used the Cochrane risk of bias assessment tool to evaluate the quality of 38 RCTs (6, 23, 26–61). Results were pooled and analyzed using RevMan 5.4 software. Regarding random sequence generation, 23 studies (6, 27, 30–33, 35–37, 39, 40, 42–49, 51, 52, 54, 55, 58–61) explicitly reported using a random number table method. Two studies (23, 38) used computer-generated random sequences. The remaining 13 studies (26, 28, 29, 34, 41, 50, 53, 56, 57) only mentioned “randomization” without specifying the method. For allocation concealment, two studies (23, 38) explicitly reported the use of sequentially numbered, sealed, opaque envelopes, while the remaining 36 studies did not describe the allocation concealment method. Due to the nature of acupuncture/moxibustion interventions, blinding of participants and practitioners was not feasible in any study, resulting in a high risk of performance bias. Concerning blinding of outcome assessment, only one study (49) explicitly reported using a single-blind design with blinding of outcome assessors. Two studies (23, 37) explicitly stated that outcome assessors were unaware of group assignments or utilized objective laboratory indicators, indirectly suggesting the possibility of blinded outcome assessment. The remaining 35 studies (6, 26–36, 38–48, 50–61) did not report whether outcome assessors were blinded. Regarding data completeness, all studies reported complete outcome data or provided reasonable explanations for minor dropouts, resulting in a low risk of bias due to incomplete data. For selective reporting and other biases, all studies pre-specified and reported their primary outcomes, with no clear selective reporting bias detected, and other potential bias sources remained unclear. Overall, the included studies demonstrated acceptable performance in random sequence generation and data completeness. However, widespread limitations existed in key areas such as allocation concealment, blinding of participants and personnel, and blinding of outcome assessment, which may impart some influence on the reliability of the findings. The risk of bias assessment results are shown in Figures 2, 3.

### 3.4 Assessment of clinical relevance

To interpret the clinical significance of our findings beyond statistical significance, we compared our pooled effect estimates against established minimal clinically important differences (MCIDs). For the TNSS, Barnes et al. (62) determined the MCID to range from 0.55 to 0.73 points on a 0–12 point scale (0–3 per symptom). Given that the majority of studies in our meta-analysis employed a 0–16 point scale (0–4 per symptom), for which a specific MCID has not been established in the literature, we performed a linear conversion, yielding an approximate MCID of 0.73–0.97 points on the 0–16 scale. This converted estimate is used only as a qualitative benchmark and not as a definitive cutoff for clinical significance. For the RQLQ, Juniper et al. (63) established the MCID as 0.5 points on the 0–6 per-item average scale. These thresholds serve as qualitative reference points for interpreting clinical relevance. However, because the pooled effect sizes in this meta-analysis are reported as SMD and the original studies did not consistently report the raw standard deviations required for back-calculation, we are unable to directly determine whether the observed raw score differences exceed these MCID thresholds. In the subsequent sections, the clinical relevance of the pooled effect sizes (reported as SMDs) will be interpreted qualitatively by comparing their magnitude against established standards (e.g., |SMD| ≥ 0.5 as moderate effect, |SMD| ≥ 0.8 as large effect), with reference to the MCID thresholds as contextual benchmarks rather than direct quantitative cutoffs.

### 3.5 Meta-analysis of the results

#### 3.5.1 Acupuncture therapy vs. conventional medication

##### 3.5.1.1 TNSS score

**FIGURE 4:**
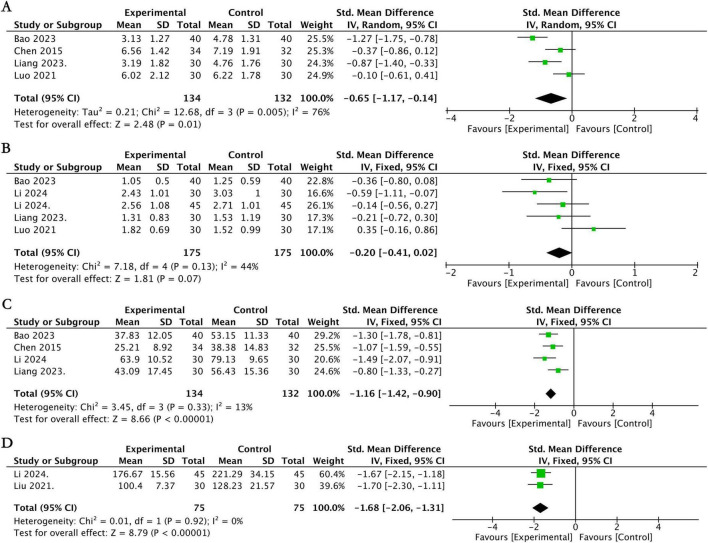
Forest plot for acupuncture therapy vs. conventional medication. **(A)** Forest plot of the TNSS score. **(B)** Forest plot of the TNNSS score. **(C)** Forest plot of the RQLQ score. **(D)** Forest plot of the total IgE level.

Among the included studies, four RCTs (6, 23, 40, 46) involving a total of 266 participants evaluated changes in nasal symptoms using the TNSS scale. The pooled analysis showed that compared with conventional medication, acupuncture therapy demonstrated a significant advantage in improving nasal symptoms in patients with AR at the end of treatment (SMD = −0.65, 95% CI [−1.17, −0.14], *P* = 0.01; *I*^2^ = 76%; Figure 4A), with a statistically significant difference between the two groups. By conventional thresholds, this effect size represents a moderate-to-large effect (|SMD| = 0.65). When qualitatively referencing the estimated MCID range for TNSS (0.73–0.97 points on the 0–16 scale), the magnitude of this effect is suggestive of a clinically perceptible improvement, though a direct quantitative comparison is not feasible due to the use of SMD.

##### 3.5.1.2 TNNSS score

Five studies (23, 37, 38, 40, 46) involving 350 participants assessed symptom changes via the TNNSS scale. The meta-analysis indicated that acupuncture therapy and conventional medication did not differ significantly in TNNSS scores (SMD = −0.20, 95% CI [−0.41, 0.02], P = 0.07; *I*^2^ = 44%; Figure 4B).

##### 3.5.1.3 RQLQ score

Four studies (6, 23, 37, 40) involving a total of 266 participants, changes in quality of life were assessed using the RQLQ. The results indicated that compared with conventional drug therapy, acupuncture therapy significantly improved disease-specific quality of life as measured by the RQLQ in patients with AR. The pooled analysis showed a significant effect size (SMD = −1.16, 95% CI [−1.42, −0.90], *p* < 0.00001), with low heterogeneity among studies (*I*^2^ = 13%; Figure 4C). By conventional thresholds, this effect size represents a large effect (|SMD| = 1.16). When qualitatively referencing the MCID for RQLQ (0.5 points on the 0–6 per-item average scale), the magnitude of this effect is strongly suggestive of a clinically meaningful improvement in quality of life, although a direct quantitative conclusion cannot be drawn from the SMD alone.

##### 3.5.1.4 Total IgE level

A total of 150 participants were enrolled in two trials (38, 42) designed to evaluate changes in total IgE levels. The meta-analysis demonstrated a statistically significant reduction in total IgE levels with acupuncture therapy compared to conventional drug therapy (SMD = −1.68, 95% CI [−2.06, −1.31], p < 0.00001; *I*^2^ = 0%; Figure 4D).

##### 3.5.1.5 Effective rate

**FIGURE 5:**
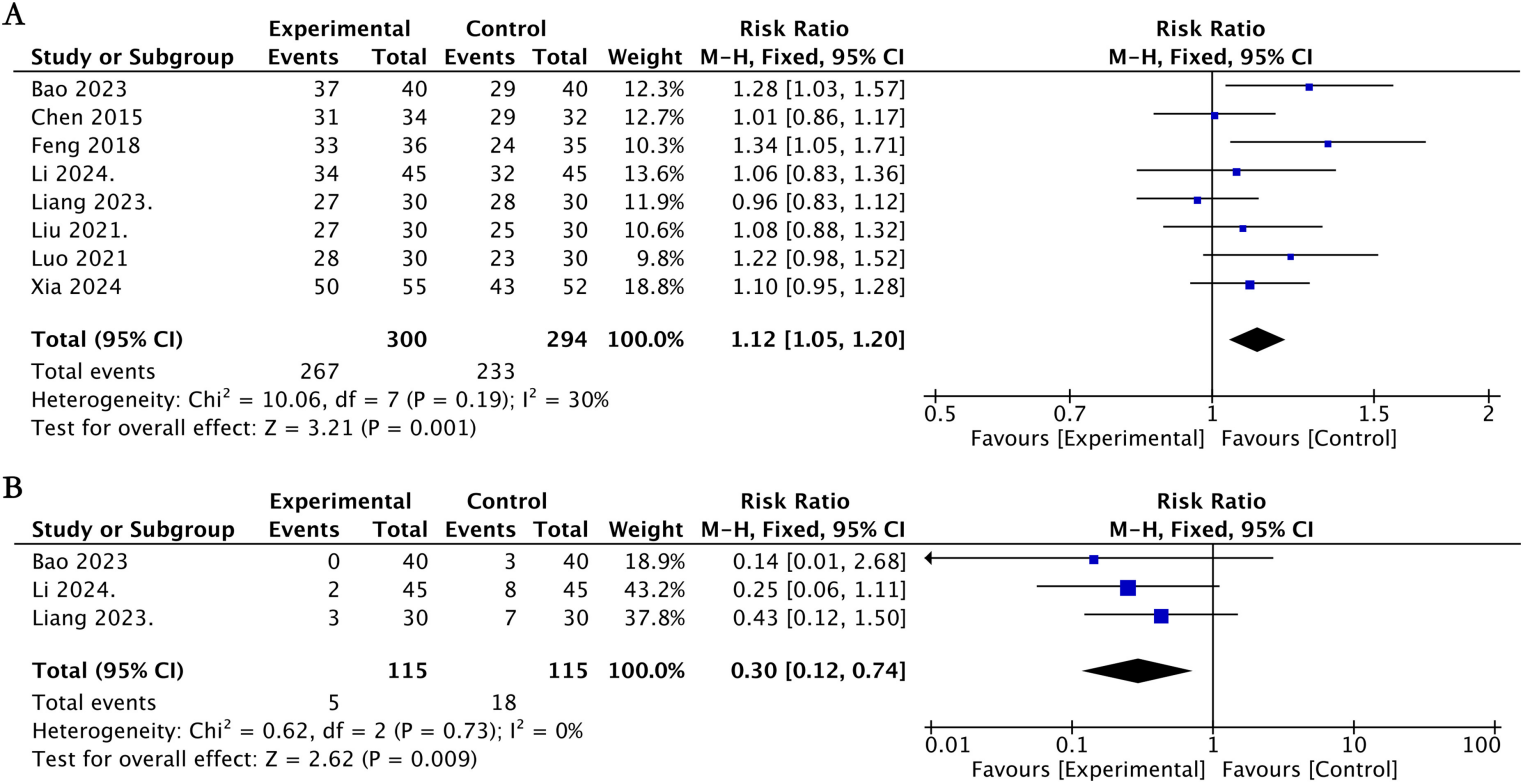
Forest plot for acupuncture therapy vs. conventional medication. **(A)** Forest plot of the effective rate. **(B)** Forest plot of the adverse event rate.

Eight studies (6, 23, 29, 38, 40, 42, 46, 55) reported the overall effective rate at the end of treatment, involving 594 patients with AR. Among them, the experimental group comprised 300 cases, while the control group had 294 cases. Heterogeneity tests indicated no significant statistical heterogeneity among these studies (*I*^2^ = 30%). The meta-analysis demonstrated that the effective rate in the group receiving acupuncture therapy was significantly higher than that in the group using conventional medication alone (RR = 1.12, 95% CI [1.05, 1.20], *P* = 0.001; Figure 5A).

##### 3.5.1.6 Adverse event rate

Four studies (23, 38, 40, 55) reported adverse events in both the acupuncture therapy and conventional medication groups. The initial meta-analysis showed no statistically significant difference in the incidence of adverse events between the two groups (RR = 0.56, 95% CI [0.15, 2.03], *P* = 0.38, *I*^2^ = 59%). The study by Xia et al. (55) was identified as the primary source of this heterogeneity. Given that this study also carried a high risk of selection and detection bias, it was excluded in a sensitivity analysis. Following its exclusion, the pooled results demonstrated a statistically significant lower incidence of adverse events in the acupuncture therapy group (RR = 0.30, 95% CI [0.12, 0.74], *P* = 0.009, *I*^2^ = 0%; Figure 5B). Common adverse events in the acupuncture therapy group included bruising, headache, and needle pain, whereas the medication group frequently reported dry nose, epistaxis, headache, drowsiness, as well as nausea. All reported adverse events were mild and self-limiting, with no serious adverse events reported.

#### 3.5.2 Acupuncture therapy plus conventional medication vs. conventional medication

##### 3.5.2.1 TNSS score

**FIGURE 6:**
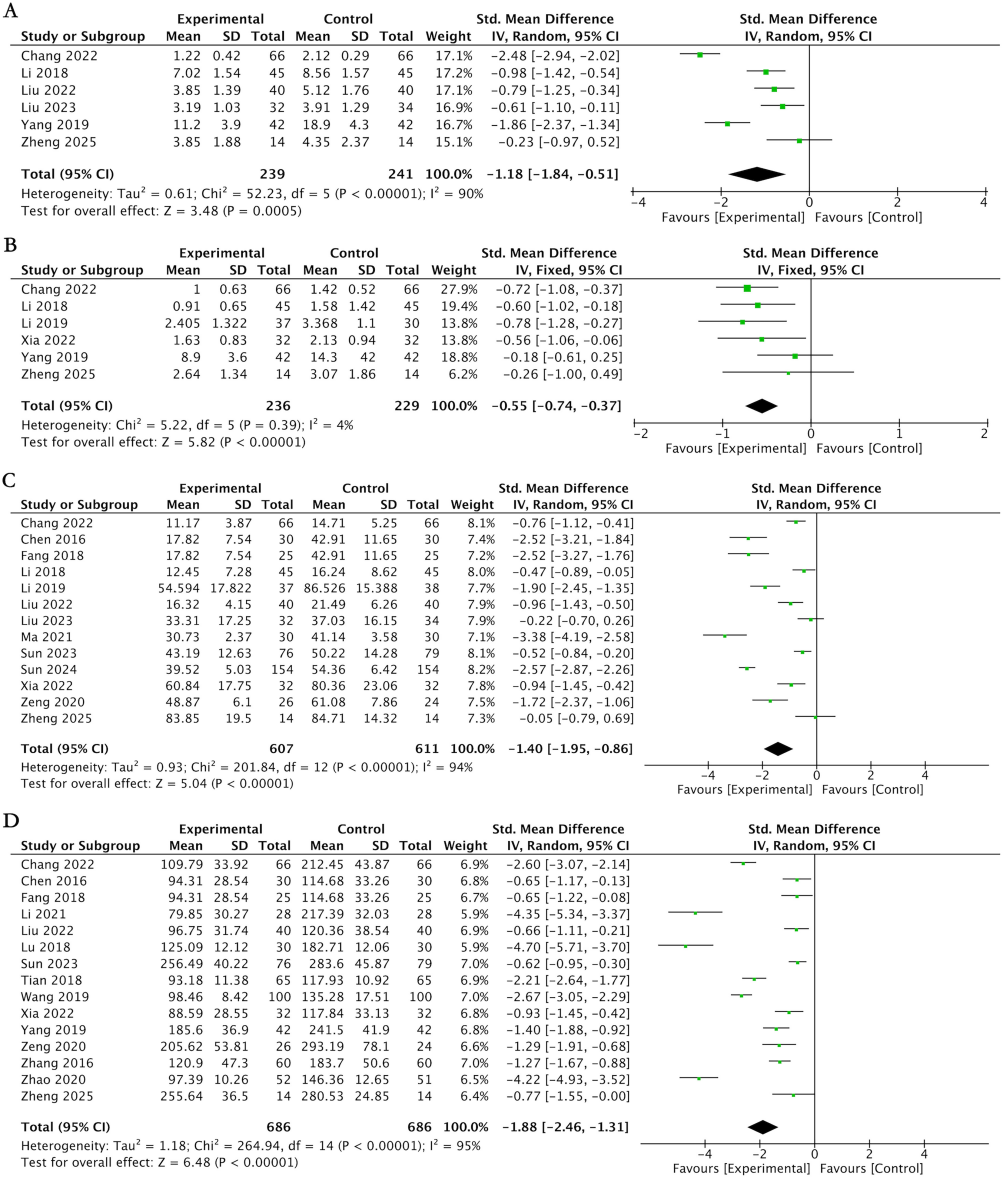
Forest plot for acupuncture therapy plus conventional medication vs. conventional medication. **(A)** Forest plot of the TNSS score. **(B)** Forest plot of the TNNSS score. **(C)** Forest plot of the RQLQ score. **(D)** Forest plot of the total IgE level.

Six studies (26, 34, 43, 44, 57, 61) utilized the TNSS scale to assess the improvement of nasal symptoms, involving 480 subjects. The pooled analysis indicated that, at the end of treatment, acupuncture therapy showed a significant advantage over conventional medication in improving nasal symptoms for patients with AR (SMD = −1.18, 95% CI [−1.84, −0.51], *P* = 0.0005; *I*^2^ = 90%; Figure 6A), and the between-group difference was statistically significant. By conventional thresholds, this effect size represents a large effect (|SMD| = 1.18). When qualitatively referencing the estimated MCID range for TNSS (0.73–0.97 points on the 0–16 scale), the magnitude of this effect suggests that the addition of acupuncture to conventional medication may provide a clinically perceptible benefit to patients, though this interpretation remains qualitative due to the absence of raw score back-calculation.

##### 3.5.2.2 TNNSS score

Six studies (26, 34, 35, 54, 57, 61) used the TNNSS scale to evaluate symptom changes, involving 465 subjects. The meta-analysis showed that acupuncture therapy was significantly superior to conventional medication in improving the TNNSS score (SMD = −0.55, 95% CI [−0.74, −0.37], *p* < 0.00001; *I*^2^ = 4%; Figure 6B).

##### 3.5.2.3 RQLQ score

Thirteen studies (26–28, 34, 35, 43, 44, 47–49, 54, 58, 61) used the RQLQ scale to assess changes in quality of life, involving 1,218 subjects. The results showed that, compared to conventional medication, acupuncture therapy significantly improved the disease-specific quality of life in AR patients. The pooled analysis indicated a significant effect size (SMD = −1.40, 95% CI [−1.95, −0.86], *p* < 0.00001; *I*^2^ = 94%; Figure 6C). By conventional thresholds, this effect size represents a large effect (|SMD| = 1.40). When qualitatively referencing the MCID for RQLQ (0.5 points on the 0–6 per-item average scale), the magnitude of this effect is strongly suggestive of a clinically meaningful improvement in quality of life. Despite the high statistical heterogeneity (*I*^2^ = 94%), the consistent pattern of large effect sizes across studies supports the robustness of this clinical finding, noting that a direct quantitative comparison with the MCID is not possible from SMDs alone.

##### 3.5.2.4 Total IgE level

Fifteen studies (26–28, 36, 43, 45, 48, 50, 51, 55, 57–61) evaluated changes in total IgE levels, involving 1,372 subjects. The meta-analysis indicated that, compared to conventional medication, acupuncture therapy significantly reduced total IgE levels (SMD = −1.88, 95% CI [−2.46, −1.31], *p* < 0.00001; *I*^2^ = 95%; Figure 6D).

##### 3.5.2.5 Effective rate

**FIGURE 7:**
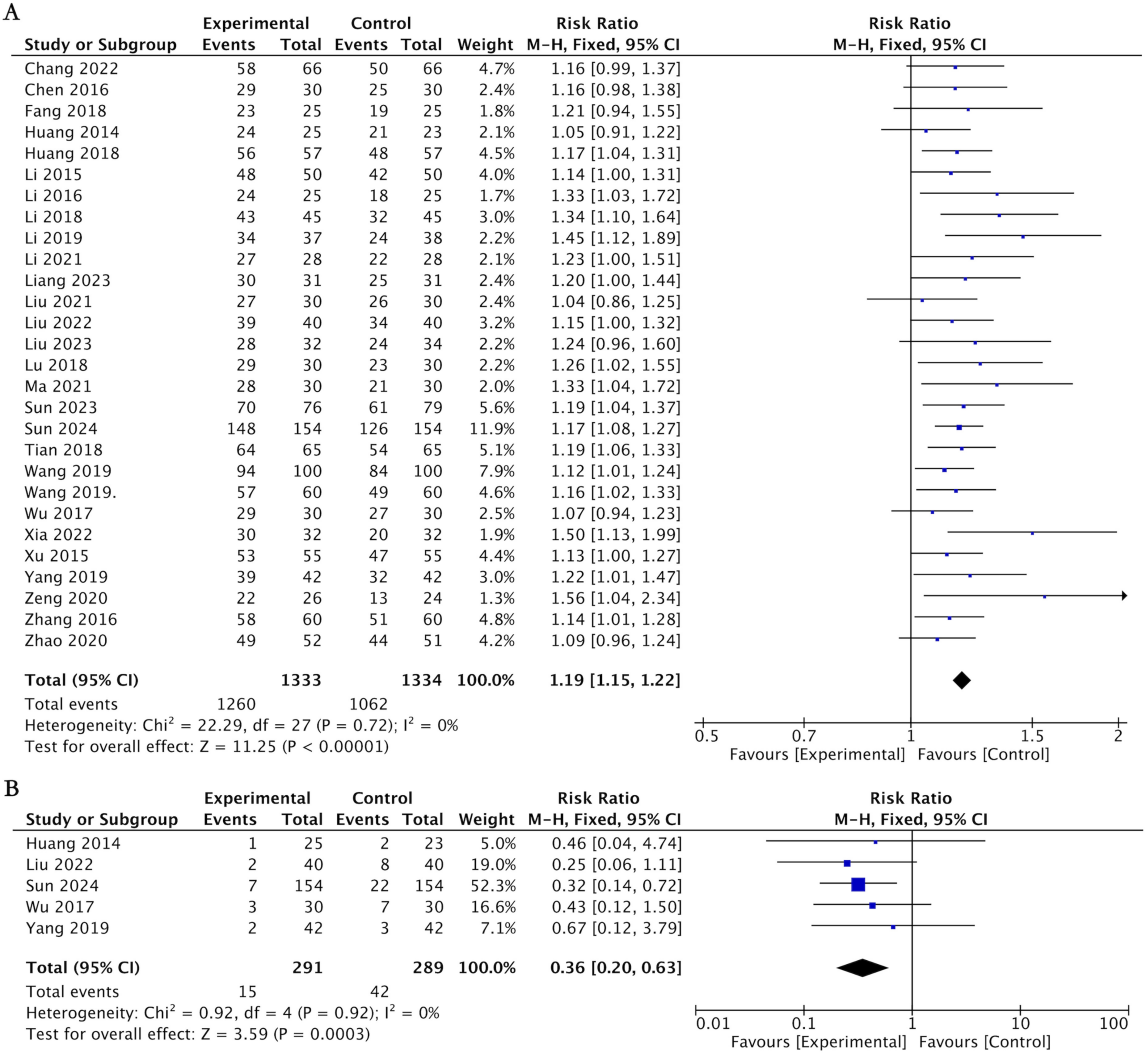
Forest plot for acupuncture therapy plus conventional medication vs. conventional medication. **(A)** Forest plot of the effective rate. **(B)** Forest plot of the adverse event rate.

Twenty-eight studies (26–28, 30–36, 39, 41, 43–45, 47–54, 56–60) reported the total effective rate at the end of treatment, including a total of 2,667 AR patients, with 1,333 in the experimental group and 1,334 in the control group. Heterogeneity testing showed no significant statistical heterogeneity among these studies (*I*^2^ = 0%). The meta-analysis demonstrated that the total effective rate in the acupuncture treatment group was significantly higher than that in the conventional medication-only group (RR = 1.19, 95% CI [1.15, 1.22], *p* < 0.00001; Figure 7A).

##### 3.5.2.6 Adverse event rate

Five studies (30, 43, 49, 53, 57) reported adverse events in both the acupuncture and medication groups, primarily mild adverse reactions such as nasal dryness, epistaxis, nausea and vomiting, and drowsiness. The pooled results showed that the incidence of adverse events in the acupuncture therapy group was significantly lower than that in the conventional medication group (RR = 0.36, 95% CI [0.20, 0.63], *P* = 0.0003; *I*^2^ = 0%; Figure 7B).

### 3.6 Acupuncture points

In the 38 included studies, a total of 60 acupoints (including extra points) were identified for the treatment of allergic rhinitis. In clinical practice, these acupoints are combined in various formulations to create treatment prescriptions. The most commonly used point combinations centered on Yingxiang (LI20) and Yintang (EX-HN3), frequently paired with points such as Shenshu (BL23), Baihui (GV20), and Dazhui (GV14) to form a treatment strategy addressing both the root cause and symptoms of allergic rhinitis. Based on their frequency of appearance across different studies, the acupoints can be categorized into the following four tiers: the most commonly used points (in > 75% of studies) were LI20 and EX-HN3; those used in 50–75% of studies (commonly used) were Hegu (LI4), Feishu (BL13), Zusanli (ST36), Fengchi (GB20), and Bitong (EX-HN9); and points used in 25–50% of studies (often used) comprised BL23, GV20, and GV14; Points with lower frequency of use (used in 10–25% of studies) included Neiyingxiang (EX-HN10), Guanyuan (CV4), Taichong (LR3), Waiguan (TE5), Pishu (BL20), Sanyinjiao (SP6), Tianshu (ST25), Lieque (LU7), Fengmen (BL12), Zhongwan (CV12), Qihai (CV6), Fengfu (GV16), Cuanzhu (BL2), Quchi (LI11), Geshu (BL17), Shangxing (GV23), Taibai (SP3), Yinlingquan (SP9), Dazhu (BL11), Sibai (ST2), and Taiyang (EX-HN5); as shown in Table 3.

**TABLE 3:** Acupuncture points selected for the treatment of allergic rhinitis.

Interventions	Frequency of use	Acupuncture points
Acupuncture	Most commonly used (in > 75% of studies)	LI20, EX-HN3
	Commonly used (in 50–75% of studies)	LI4, BL13, ST36, GB20, EX-HN9
	Often used (in 25–50% of studies)	BL23, GV20, GV14
	Sometimes used (in 10–25% of studies)	EX-HN10, CV4, LR3, TE5, BL20, SP6, ST25, LU7, BL12, CV12, CV6, GV16, BL2, LI11, BL17, GV23, SP3, SP9, BL11, ST2, EX-HN5

The number and selection of acupoints varied across treatment protocols, reflecting the characteristic flexibility in clinical acupuncture practice based on individual pattern differentiation. The combination of core and primary acupoints forms the foundation of the prescription, while auxiliary and syndrome-differentiation acupoints are added or subtracted according to the patient’s specific pattern type and accompanying symptoms.

From the perspective of traditional Chinese medicine theory, the combination of high-frequency acupoints adheres to the therapeutic principles of “combining local and distal point selection” and “integrating organ-system regulation with meridian regulation.” LI20, located on the nasolabial groove beside the ala of the nose, and EX-HN9, situated at the junction of the ala of the nose and the nasolabial groove, are both located near the nose, representing local point selection. They act directly on the affected orifice to dispel stagnation and promote the free flow of Qi in the nasal passage, serving as rapid interventions for excess patterns such as nasal congestion and discharge. EX-HN3 is located on the forehead, at the midpoint between the eyebrows, along the Governor Vessel (Du Mai) pathway which connects to the brain. It can calm the mind and regulate the Yang Qi of the Du Mai. This helps alleviate the forehead discomfort and mental tension often associated with the condition.

Distal point selection focuses on regulating the causative factors and pathological mechanisms. LI4, located on the dorsum of the hand between the 1st and 2nd metacarpal bones, is the Yuan-Source point of the Large Intestine Meridian of Hand-Yangming (whose pathway ascends to the nose). It can disperse wind, clear heat, and regulate Qi and blood flow in the facial region. This is a classic application of the principle “where the meridian passes, it can treat.” BL13, located 1.5 cun lateral to the lower border of the spinous process of the 3rd thoracic vertebra, is the Back-Shu point of the Lung (Fei). It directly tonifies Lung (Fei) Qi and strengthens the defensive exterior, aiming to address the internal root cause of “Lung (Fei) Qi deficiency and cold, leading to weakened defensive capacity.” ST36, located 3 cun below Dubi (ST35), one finger’s breadth lateral to the anterior crest of the tibia, is the He-Sea point of the Stomach Meridian and a major tonic point. It functions to strengthen the Spleen and Stomach (Pi Wei), and supplement Qi and Blood. It supports Lung (Fei) Qi by reinforcing the “Earth” to generate the “Metal,” thereby consolidating the acquired constitution. GB20, located in the depression between the upper portion of the sternocleidomastoid and the trapezius muscles, is a key point for expelling wind. It is particularly effective in dispersing externally-contracted wind pathogens, intercepting the external pathway that triggers the condition. Together, these acupoints create a multi-layered therapeutic system that operates from local to systemic levels, from expelling pathogens to supporting the body’s righteous Qi, and from symptom control to constitutional regulation. This exemplifies the concrete application of syndrome differentiation, treatment based on pattern identification, and the holistic concept in the selection of acupuncture points.

### 3.7 Adverse reactions

Nine clinical studies (23, 30, 38, 40, 43, 49, 53, 55, 57) reported treatment-related adverse reactions, which primarily consisted of local nasal symptoms (such as nasal dryness, epistaxis, and nasal pain or irritation), gastrointestinal symptoms (such as nausea, vomiting, and gastric discomfort), and neurological manifestations (such as headache, dizziness, drowsiness, and fatigue). Other less frequently reported adverse events included bruising at acupuncture sites, dry mouth, and isolated cases of needling pain. These reactions occurred across different treatment regimens (e.g., acupuncture, nasal corticosteroids, and oral antihistamines), with their frequency and type varying by intervention.

Meta-analysis results revealed low heterogeneity among the included studies (*I*^2^ = 13%, *p* = 0.32), suggesting good consistency in the findings. The pooled analysis showed a significantly lower incidence of adverse events in the experimental group compared to the control group [RR = 0.44, 95% CI (0.28, 0.67); Supplementary Figure S1]. The test for overall effect was statistically significant (*p* = 0.0002), indicating that the experimental treatment regimens may have a potential advantage in terms of safety, as shown in Table 4.

**TABLE 4:** Adverse reaction of the included studies on acupuncture-related therapies in allergic rhinitis.

Study	Reported adverse reactions
Bao 2023 (23)	Acupuncture: No adverse reactions. Budesonide nasal spray: three cases of nasal dryness.
Huang 2014 (30)	Acupuncture+Fluticasone propionate nasal spray: one case of nasal dryness. Fluticasone propionate nasal spray: two cases (one case of nasal dryness and one case of epistaxis).
Li 2024 (38)	Acupuncture: two cases (one case of epistaxis and one case of needling pain). Mometasone furoate nasal spray: eight cases (two cases of headache, two cases of epistaxis, and four cases of dry nose and pain or irritation in the nasal area).
Liang 2023 (40)	Acupuncture : three cases of bruising at the abdominal acupoint sites (resolved spontaneously within 2–7 days). Loratadine: seven cases (six cases of drowsiness and one case of nausea and discomfort).
Liu 2022 (43)	Acupuncture+Mometasone furoate nasal spray: two cases (one case of nausea and vomiting and one case of dizziness). Mometasone furoate nasal spray: eight cases (five cases of nausea and vomiting, two cases of fatigue, and one case of dizziness).
Sun 2024 (49)	Warm acupuncture+Fluticasone propionate nasal spray: seven cases (three cases of nasal mucosal dryness, two cases of epistaxis, one case of drowsiness, and one case of nausea and vomiting). Fluticasone propionate nasal spray: twenty-two cases (six cases of nasal mucosal dryness, seven cases of epistaxis, five cases of drowsiness, and four cases of nausea and vomiting).
Wu 2017 (53)	Acupuncture+Loratadine/Budesonide nasal spray: three cases of dry mouth. Loratadine+Budesonide nasal spray: seven cases (two cases of fatigue, one case of headache, two cases of drowsiness, one case of nausea, and one case of vomiting).
Xia 2024 (55)	Acupuncture: two cases of headache (withdrew from the trial). Fluticasone propionate nasal spray: seven cases of minor local epistaxis (stopped spontaneously without special treatment).
Yang 2019 (57)	Warm acupuncture+Ebastine/Budesonide nasal spray: two cases (one case of headache and one case of gastric discomfort). Ebastine+Budesonide nasal spray: three cases (one case of gastric discomfort and two cases of dry mouth).

### 3.8 Heterogeneity analysis

In the meta-analysis, some results showed heterogeneity. Analysis of the original data suggested potential methodological heterogeneity, mainly due to insufficient description of blinding and allocation concealment in the included studies. At the same time, clinical heterogeneity could be caused by factors such as the inclusion population, acupuncture points, and operation methods. However, due to the lack of detailed descriptions of these factors in the original studies and the small number of studies in some results, further exploration of the source of heterogeneity through subgroup analysis was not possible. We conducted sensitivity analyses by sequentially excluding each study associated with significant heterogeneity (*I*^2^ ≥ 50%). For the comparison of acupuncture therapy vs. conventional medication on TNSS score (*I*^2^ = 76%), excluding any single study did not fundamentally change the direction or significance of the pooled effect, and the I^2^ value remained high. This indicates that the results are stable, and the heterogeneity likely stems from clinical or methodological diversity rather than from any single influential study. In the comparison of adverse event rates, excluding the study by Xia et al.(55) reduced the I^2^ from 59 to 0%, confirming that this study was the main source of heterogeneity. Similarly, for the comparison of TNSS score (*I*^2^ = 90%) and RQLQ score (*I*^2^ = 94%) in the combination therapy group (acupuncture therapy plus conventional medication vs. conventional medication), sensitivity analysis confirmed that the results were stable, with the effect estimates remaining consistent across iterations. Therefore, a random-effects model was adopted in all meta-analyses, and the results obtained are considered reliable.

### 3.9 Publication bias

**FIGURE 8:**
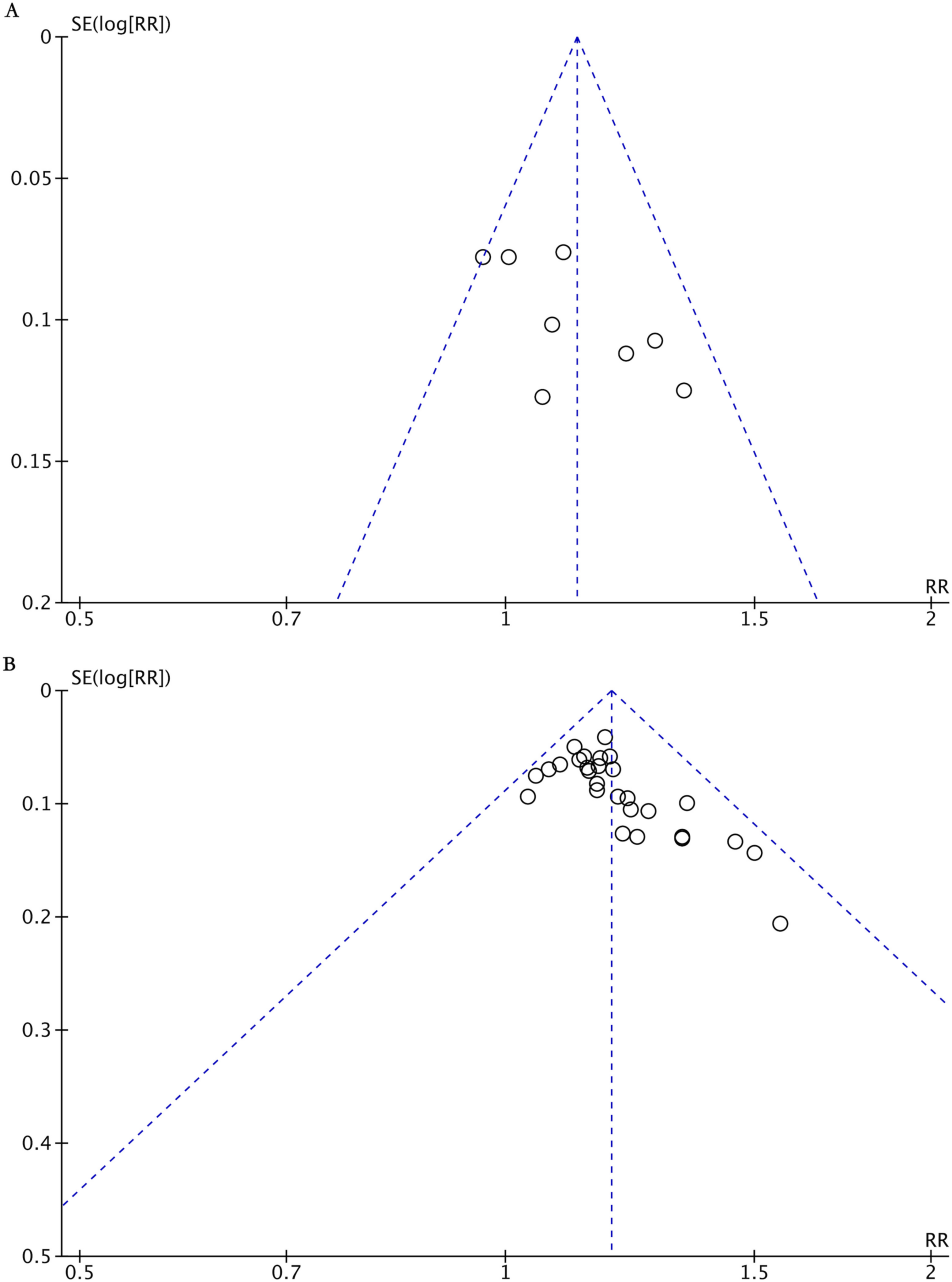
Funnel plot of effective rate. **(A)** Acupuncture therapy vs. conventional medication. **(B)** Acupuncture therapy plus conventional medication vs. conventional medication.

The funnel plot of the overall treatment effective rate was used to assess publication bias in this study. The results indicated that a certain degree of publication bias was only observed in the comparison between acupuncture therapy and conventional medication. This may be attributed to the underreporting or non-publication of negative results from small-scale studies, which should be taken into consideration when interpreting the findings (Figure 8).

## 4 Discussion

This systematic review evaluated treatment approaches combining acupuncture with conventional medication, including a total of 38 studies with 3,349 participants. We comprehensively assessed the effectiveness and safety of acupuncture alone or in combination with conventional medication for AR. Compared with conventional medication, low-certainty evidence suggests that acupuncture therapy provides minor benefits in relieving AR symptoms and improving quality of life. In terms of immunological markers, acupuncture reduced total IgE levels and was associated with fewer adverse events. When comparing acupuncture therapy combined with conventional medication to conventional medication alone, the results indicate that the combined treatment not only effectively alleviates nasal symptoms and enhances quality of life in AR patients but also demonstrates significant benefits in regulating immune markers, notably reducing total IgE levels. Additionally, the incidence of adverse events was significantly lower with the combined therapy compared to conventional medication alone, with no serious harm reported. Beyond statistical significance, we qualitatively referenced MCID thresholds to assess clinical relevance. Although direct quantitative comparison was not feasible, the effect sizes for TNSS (SMD = −0.65 to −1.18) and RQLQ (SMD = −1.16 to −1.40) fell within the moderate-to-large range, suggesting clinically perceptible benefits from acupuncture-related therapies, consistent with their significantly lower adverse event rates.

Compared with previously published systematic reviews, the core findings of this study are generally consistent in direction with those of Yin et al. (2020, 39 studies) (64) and Lin et al. (2026, 56 studies) (65). However, prior reviews have largely focused on comparisons between acupuncture alone versus sham acupuncture or between different acupuncture techniques, whereas our study explicitly focuses on the comparison of “acupuncture plus conventional medication versus conventional medication alone.” Furthermore, in contrast to He et al. (2022, 30 studies) (66), who found no clear advantage of acupuncture over conventional medication, our study demonstrates that combination therapy is significantly superior to medication alone across multiple outcomes, providing a clearer clinical positioning of acupuncture as an adjunctive treatment. Chen et al. (67) conducted a network meta-analysis to further compare the efficacy of different acupuncture techniques, aiming to explore which acupuncture technique is superior, while our study provides specific evidence on the effectiveness and safety of combination therapy based on available data. In summary, this study is the first to systematically evaluate and confirm the add-on effectiveness and safety of acupuncture combined with conventional medication compared with medication alone, quantitatively reporting the advantages of combination therapy in reducing total IgE levels and decreasing adverse events, thereby addressing the gaps in immunological outcomes and safety comparisons in previous reviews. However, the quality of the evidence remains relatively low, primarily due to persistent and non-negligible risks of bias in the included studies. A key source of bias is the inherent difficulty in achieving complete blinding of participants and personnel in acupuncture RCTs, which may introduce performance bias. Furthermore, inadequate reporting of allocation concealment and selective outcome reporting in several trials further compromise the overall certainty of the evidence. Therefore, caution is warranted when interpreting these findings and applying them to clinical decision-making.

### 4.1 Effectiveness and safety of acupuncture therapy compared with conventional medication

Multiple clinical studies have demonstrated that acupuncture therapy alone is more effective than conventional medication in improving nasal symptoms, physical signs, and quality of life in patients with AR (68, 69). In terms of symptom scores, acupuncture not only significantly alleviates individual symptoms such as nasal congestion, nasal itching, rhinorrhea, and sneezing but also markedly reduces the TNSS, reflecting its strong overall symptom control capability. The mechanism of action may involve multi-pathway regulation: on one hand, by modulating autonomic nervous function and inhibiting abnormal parasympathetic excitation, it promotes local vasoconstriction and reduces glandular hypersecretion (70, 71); on the other hand, acupuncture can also regulate serum-specific IgE, eosinophil levels, and various inflammatory cytokines, correcting the imbalance at the immune level and alleviating allergic inflammatory responses (72, 73), demonstrating the advantage of multi-target intervention. In terms of safety, adverse reactions to acupuncture treatment for AR are relatively mild, primarily manifesting as transient local soreness or minor bleeding, with rare serious adverse events. Overall, it has high safety and good clinical applicability (74).

### 4.2 Effectiveness and safety of acupuncture therapy combined with conventional medication versus medication alone

Acupuncture therapy combined with conventional medication for the treatment of AR demonstrates a clear synergistic effect, showing superior outcomes over medication alone in terms of symptom control, immunoregulation, and reduction of recurrence rates. Multiple clinical studies have confirmed that the combined therapy can more significantly improve patients’ TNSS and RQLQ scores (75).

Regarding laboratory indicators, the combined therapy exhibits advantages in multi-target regulation: it can more effectively reduce serum IgE, eosinophil count, as well as levels of interleukins (IL-4, IL-6, IL-13) and vascular cell adhesion molecule-1 (VCAM-1); simultaneously, it increases the expression of interferon-gamma (IFN-γ) and transforming growth factor-beta (TGF-β), thereby correcting the Th1/Th2 immune imbalance and inhibiting the release of inflammatory mediators (76–79). For example, a study by Zhao et al. (60) showed that acupuncture at the sphenopalatine ganglion combined with loratadine significantly reduced IgE levels and effectively improved patients’ clinical symptoms. The mechanism of action involves multi-pathway regulation: on one hand, acupuncture activates the sympathetic nerves, promoting vasoconstriction in the nasal mucosa and alleviating local congestion and edema; on the other hand, it inhibits parasympathetic nerve activity and the release of sensory neuropeptides (such as Substance P), thereby reducing glandular secretion and inflammatory cell infiltration (80, 81). Furthermore, therapies such as warm acupuncture combined with conventional medication can also exert effects of “warming the meridians, unblocking collaterals, reinforcing healthy qi, and consolidating the exterior,” thereby enhancing the body’s immunity and reducing dependence on medication and related adverse reactions (82). In terms of safety, the combined treatment regimen does not increase the incidence of adverse events. Some studies further indicate that this therapy helps reduce the dosage of antihistamines or hormones, thereby lowering the risk of drug-related side effects (83).

### 4.3 Mechanism of action of acupuncture in treating AR

The mechanism of action of acupuncture in treating AR involves multiple regulatory pathways encompassing the nervous, immune, and endocrine systems. Contemporary medicine posits that the pathogenesis of AR is closely related to IgE-mediated type I hypersensitivity, Th1/Th2 cell imbalance, eosinophil infiltration, and neurological dysfunction (84, 85). By stimulating specific acupoints (such as the sphenopalatine ganglion, LI20, BL13, etc.), acupuncture effectively regulates the dynamic balance between the sympathetic and parasympathetic nerves, suppressing excessive parasympathetic excitation. This consequently reduces the release of inflammatory mediators like histamine and leukotrienes, alleviating symptoms such as nasal congestion and rhinorrhea (86). Regarding immunomodulation, acupuncture can downregulate serum IgE levels, peripheral blood eosinophil counts, and Th2-type cytokines (IL-4, IL-5, IL-13), while upregulating Th1 cytokines (IFN-γ) and anti-inflammatory factors (TGF-β, IL-10). Thereby, it corrects the Th1/Th2 immune imbalance and inhibits mast cell degranulation and the activation and migration of eosinophils (87–89). Furthermore, acupuncture can mitigate inflammatory response and tissue damage in the nasal mucosa through multiple targets by modulating the Toll-like receptor (TLRs) and nuclear factor-kappa B (NF-κB) signaling pathways, as well as the expression of Transient Receptor Potential Vanilloid 1 (TRPV1) (90, 91).

From the perspective of TCM theory, AR falls under the category of “Bi Qiu”. Its pathogenesis is rooted in qi deficiency of the Lung (Fei), Spleen (Pi), and Kidney (Shen); with invasion by wind, cold, and dampness pathogens as the superficial manifestations. Acupuncture therapy, by stimulating meridians and acupoints, functions to unblock the meridians, reinforce healthy qi, and expel pathogenic factors. It achieves therapeutic effects of diffusing the Lung (Fei) and unblocking the orifices, as well as boosting qi and consolidating the exterior, thereby improving the patient’s constitution and reducing recurrence (92, 93).

### 4.4 Acupuncture treatment regimen

A comprehensive analysis of the included literature revealed considerable variability in the selection of acupuncture points across studies, encompassing a total of 60 acupoints (including extra points). Among these, LI20 and EX-HN3 were the most frequently used (appearing in > 75% of studies), followed by points such as LI4, BL13, ST36, GB20, and EX-HN9 (appearing in 50–75% of studies). Additionally, points including BL23, GV20, GV14, as well as EX-HN10, CV4, and LR3, were often selected flexibly based on pattern differentiation.

This diversity in point selection reflects the personalized therapeutic approach inherent in TCM, known as “treating the same disease with different methods.” In TCM theory, while allergic rhinitis manifests with nasal symptoms, its root pathogenesis is closely linked to the functional state of the zang-fu organs, particularly the Lung (Fei), Spleen (Pi), and Kidney (Shen). The Lung (Fei) opens into the nose, governs Qi and respiration, and is associated with the skin and body surface. Lung (Fei) Qi deficiency and weakness of the defensive (Wei) Qi make the body susceptible to invasion by external pathogens such as wind, cold, and dampness, leading to nasal obstruction, sneezing, and clear rhinorrhea. The Spleen (Pi) is the source of Qi and blood production and governs transportation and transformation. Spleen (Pi) deficiency results in insufficient Qi and blood, failing to nourish the Lung (Fei) and defensive systems; moreover, impaired fluid metabolism leads to phlegm accumulation, which ascends to obstruct the nasal passages, exacerbating congestion and rhinorrhea. The Kidney (Shen) is the foundation of congenital constitution, governs reception of Qi, and stores essence. Kidney (Shen) Qi deficiency leads to inadequate warming and motivating capacity, thereby weakening Lung (Fei) and Spleen (Pi) Qi. It may also result in upward disturbance of cold fluids, contributing to persistent nasal symptoms aggravated by cold exposure. Therefore, treatment should not merely focus on local nasal stimulation but must address the underlying disharmony of zang-fu functions from a holistic perspective.

Despite the variability in specific point combinations, a relatively stable and frequently applied treatment framework emerged from the literature: core local points around the nose (e.g., LI20, EX-HN3) to quickly unblock the nasal orifice and alleviate symptoms, combined with distal and systemic regulating points (e.g., LI4, BL13, ST36, GB20) to reinforce healthy qi and regulate the zang-fu organs. This framework embodies the integrated TCM principles of “local point selection” (treating the area where the point is located) and “distal point selection along the meridian” (treating conditions along the pathway of the meridian). More importantly, it incorporates the fundamental concept of “simultaneously addressing the root and branch”: treating the branch involves opening the nasal orifice and dispelling pathogenic factors, while treating the root involves tonifying Lung (Fei) Qi, strengthening the Spleen and Stomach (Pi Wei), and warming Kidney (Shen) Yang. Specifically, LI4 as the Yuan-Source point of the Hand Yangming Large Intestine Meridian whose pathway ascends to the nose, is effective in dispersing wind, clearing heat, and regulating Qi and blood circulation in the facial region. BL13, the Back-Shu point of the Lung (Fei), directly tonifies Lung (Fei) Qi and strengthens the defensive exterior, targeting the Lung (Fei) as the fundamental root. ST36, the He-Sea point of the Stomach Meridian and a major tonic point, strengthens the Spleen and Stomach (Pi Wei), supplements Qi and Blood, and supports Lung (Fei) Qi through the “reinforcing Earth to generate Metal” principle. GB20 is a key point for expelling wind, particularly effective in dispersing external wind pathogens that invade the nasal orifice. Together, these points establish a multi-layered therapeutic system operating from local to systemic levels, from dispelling pathogens to supporting healthy qi, and from symptom control to constitutional regulation. It is noteworthy that needling points in delicate and neurovascularly rich areas such as the nose (e.g., LI20, EX-HN9, EX-HN10) requires precise localization and technique to ensure both effectiveness and safety. Similarly, strict attention to depth and angle of insertion is necessary for frequently used points in the cervical region, such as GB20 and GV14.

In summary, despite flexibility in specific prescriptions, a clear consensus exists in the literature regarding the selection of core acupoints and the therapeutic strategy of “combining local and distal points” and “simultaneously treating the root and branch.” This balance between adhering to common therapeutic principles and implementing individualized treatment precisely demonstrates the systematic strength of acupuncture therapy in restoring the body’s Yin-Yang balance and regulating Qi and blood status. It also reflects its patient-centered nature and capacity to dynamically adapt to clinical complexity.

### 4.5 Advantages of combining acupuncture therapy with conventional medication

The combination of acupuncture and conventional medication for treating AR exemplifies the advantages of integrating traditional Chinese and Western medicine. This approach not only rapidly controls symptoms but also regulates the body’s immune status, thereby reducing drug dosage and side effects. Conventional medications (such as antihistamines and intranasal corticosteroids) typically act quickly with well-defined targets, making them suitable for acute-phase intervention. However, long-term use alone may lead to drug tolerance and potential local or systemic adverse reactions. Acupuncture, on the other hand, works through holistic regulation, improving the nasal mucosal microenvironment and neural regulatory functions, thereby enhancing the body’s adaptive capacity to antigenic stimuli and helping to prolong the duration of therapeutic effects (94, 95). Combined therapy is particularly suitable for moderate-to-severe, persistent AR patients, significantly increasing the treatment response rate and effectively improving quality of life and long-term prognosis (96, 97). Furthermore, acupuncture therapy is characterized by its operational flexibility, minimal side effects, and high patient acceptance, offering more options for the long-term management and personalized treatment of AR (98).

### 4.6 Limitations

This study has several limitations: First, the methodological quality of some included RCTs is not high, with inadequate implementation of randomization, allocation concealment, and blinding, which may affect the reliability of the results. Second, there is heterogeneity in acupuncture protocols (acupoint selection, treatment duration) and medication types across the studies, potentially introducing clinical differences when pooling the results. Third, some studies had small sample sizes and short follow-up periods, failing to adequately assess long-term effectiveness and safety. Fourth, all included studies lacked sham acupuncture control groups, which limits our ability to conclusively determine the specific efficacy of acupuncture beyond placebo effects. Therefore, our findings should be interpreted as evidence of effectiveness in clinical practice rather than specific efficacy under ideal conditions. Fifth, due to limitations in the original data, it was not possible to conduct subgroup analyses for all acupuncture-related therapies. Sixth, all of the included trials were conducted in China. Due to potential differences in genetic background, environmental allergen exposure, concomitant medication use, and acupuncture practices and training, this geographical limitation may affect the generalizability of our findings to other populations and healthcare settings. Finally, publication bias may exist, which could affect the robustness of the conclusions. Specifically, the search period was limited to studies published between 2014 and 2025. Although this restriction ensured the inclusion of recent studies that reflect contemporary clinical practice, it may have excluded older but still potentially relevant randomized controlled trials. Future research should involve more high-quality, large-sample, multi-center RCTs to further validate the efficacy and safety of acupuncture and its combination with medication for treating AR, and to further explore its mechanisms of action.

## 5 Conclusion

Acupuncture, whether administered as a standalone intervention or in combination with conventional medication, may represent an effective and safe treatment option for improving symptoms and quality of life in patients with AR. Specifically, traditional manual acupuncture and acupuncture combined with moxibustion may be superior to medication alone in controlling symptoms. Meanwhile, the combination of acupuncture as an adjunctive therapy with conventional medication may yield better effectiveness than either intervention alone, without increasing the risk of additional adverse reactions. Based on the current evidence, it is recommended that acupuncture can be reasonably considered as a treatment option for AR in clinical practice, particularly for patients seeking non-pharmacological therapies or those with suboptimal responses to medication. However, interpretation of these findings should remain cautious due to the considerable methodological heterogeneity among the included studies and the generally low certainty of evidence. Future research in this field urgently requires more methodologically rigorous, large-sample, multi-center RCTs with long-term follow-up to provide higher-quality evidence and further clarify the long-term effects of acupuncture.

## Data Availability

The data analyzed in this study is subject to the following licenses/restrictions: no new primary data were generated in this study. The analyzed data are from previously published studies, and access is governed by the original publishers’ policies. Therefore, no dataset-specific restrictions apply. Requests to access these datasets should be directed to Since this study did not generate a new primary dataset and instead analyzed data from previously published studies, no centralized dataset is available for direct request. For researchers seeking individual participant data from the original studies included in this meta-analysis, requests should be directed to the corresponding authors of the respective original publications cited in the reference list.

## Supplementary material

Supplementary Figure S1 Forest plot of the meta-analysis for adverse events.
