# Supplementary figures and images for: Efficacy and safety of acupuncture-related therapies in the treatment of allergic rhinitis: a systematic review and meta-analysis
Source: Front Med (Lausanne). 2026 May 29;13:1835311. doi: 10.3389/fmed.2026.1835311 (PMC13231950; doi:10.3389/fmed.2026.1835311)

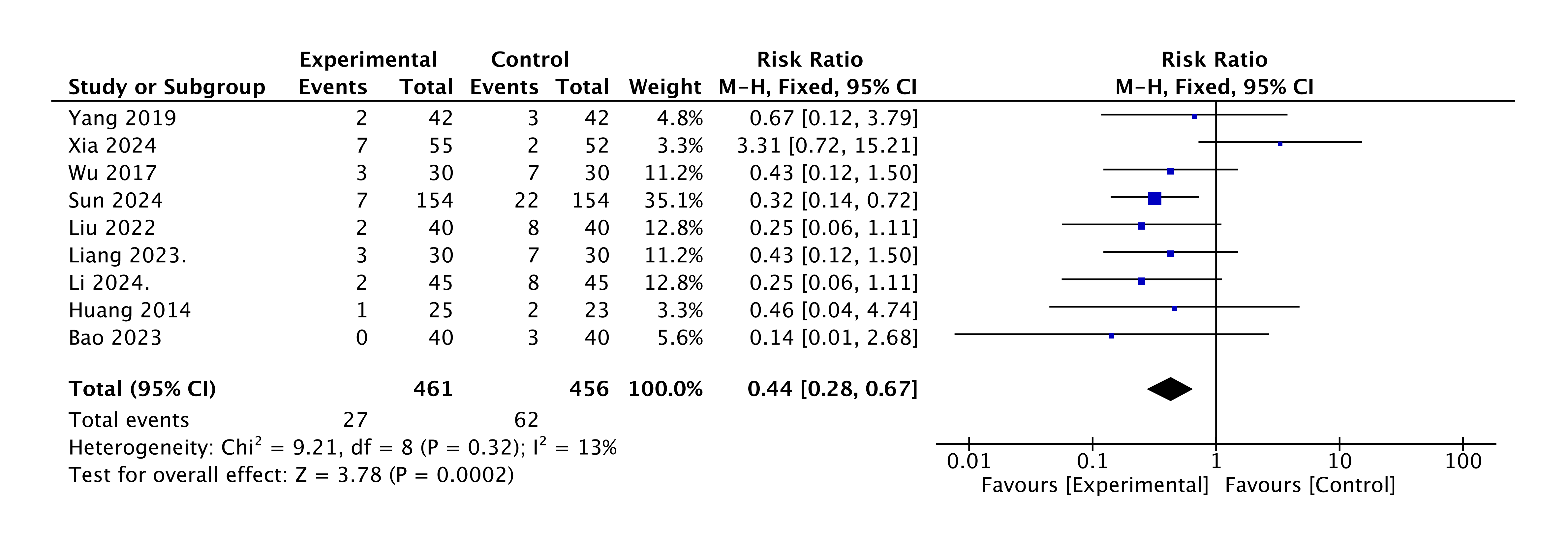

Supplement: Supplementary Figure S1 — Forest plot of the meta-analysis for adverse events. [file Image_1.tif]
